# Post-transplant Complication With TAFRO Features in a Patient With Acute Myeloid Leukemia

**DOI:** 10.7759/cureus.23688

**Published:** 2022-03-31

**Authors:** Kyosuke Yamaguchi, Yasushi Kubota, Hiroo Katsuya, Toshihiko Ando, Shinya Kimura

**Affiliations:** 1 Department of Internal Medicine, Saga University Faculty of Medicine, Saga, JPN; 2 Department of Transfusion Medicine and Cell Therapy, Saitama Medical Center, Saitama Medical University, Kawagoe, JPN

**Keywords:** multicentric castleman disease, graft-versus-host disease, tafro syndrome, cord blood, allogeneic hematopoietic stem cell transplantation

## Abstract

Thrombocytopenia, anasarca, fever, reticulin fibrosis, renal insufficiency, and organomegaly (TAFRO) syndrome was first reported in 2010 and can occur in association with various potential causes including idiopathic multicentric Castleman disease, infectious diseases, malignancies, and rheumatologic disorders. The diagnostic criteria do not mention a possible association with hematopoietic stem cell transplantation. Here, we present a 56-year-old man who had TAFRO syndrome-like complications after cord blood transplantation (CBT) for acute myeloid leukemia. At two years and seven months after CBT, he was admitted to our hospital with fever, thrombocytopenia, renal insufficiency, and elevated levels of bilirubin and C-reactive protein. Computed tomography images showed bilateral pleural effusion, pelvic ascites, and abdominal lymphadenopathy. Although his symptoms met the diagnostic criteria for TAFRO syndrome, graft-versus-host disease (GVHD) was first suspected, and he was treated with steroid pulse therapy, which was ineffective. The second line of treatment was tocilizumab as a treatment for TAFRO syndrome, which was effective to a certain extent; however, he died two years and 10 months after CBT. This is the first case report of post-transplant complications with TAFRO features, which provides a background for further research into the relationship between post-transplant TAFRO symptoms and GVHD.

## Introduction

TAFRO syndrome was first reported in 2010 and is a constellation of symptoms including thrombocytopenia, anasarca, fever, reticulin fibrosis, renal insufficiency, and organomegaly [1,2]. TAFRO syndrome is a systemic inflammatory disorder that can occur in association with various potential causes including idiopathic multicentric Castleman disease (iMCD), infectious diseases, malignancies, and rheumatologic disorders [3]. These symptoms are occasionally observed in patients who received allogeneic hematopoietic cell transplantation (allo-HCT). Thus, distinguishing TAFRO syndrome from other causes including graft-versus-host disease (GVHD) is difficult in the post-transplant setting. Here, we present the patient who showed TAFRO syndrome-like complications after cord blood transplantation (CBT).

## Case presentation

A 56-year-old man was diagnosed with acute myeloid leukemia (AML-NOS) with a normal karyotype. The patient achieved complete remission after induction therapy and subsequent consolidation therapy; however, he relapsed 11 months after the diagnosis and received re-induction therapy. Then, he received CBT from a female donor with human leukocyte antigen (HLA) 4/8 allele match at -A, -B, -C, and -DRB1 in both the GVH and HVG directions. The GVHD prophylaxis included tacrolimus and methotrexate at a dose of 5 mg/m^2^ on days 1, 3, and 6. After transplantation, the patient developed fever and skin rash on day 13 and was treated with methylprednisolone (mPSL) 1 mg/kg daily, which was tapered rapidly. Neutrophil and platelet engraftments were achieved on days 17 and 39, respectively. The patient’s course was complicated by stage 2 skin acute GVHD on day 27 and cytomegalovirus (CMV) antigenemia on day 33. He was discharged on day 86 without active GVHD. Although AML was in remission, the patient was re-admitted to our hospital because of viral hemorrhagic cystitis and disseminated adenovirus (ADV) infection on day 259 and was discharged on day 290. He suffered limited chronic localized GVHD with skin involvement and hepatic dysfunction; immunosuppressive agents were successfully discontinued on day 591.

At two years and seven months after transplantation, the patient developed enteritis with mild mediastinal lymphadenopathy and was treated with antibiotics. Stool culture showed no specific bacteria. Although diarrhea improved, he was admitted to the hospital with fever, thrombocytopenia, renal impairment, and increased bilirubin 945 days after CBT. He had no pain or polyneuropathy. Skin rash, vomiting, and diarrhea were not observed. White blood cell count, absolute neutrophil count, and absolute lymphocyte count were normal (8.6 × 10^9^/L, 6.07 × 10^9^/L, and 1.47 × 10^9^/L, respectively). He had thrombocytopenia with no morphological atypical platelets (PLT 65 × 10^9^/L), renal insufficiency (UN 17.96 mmol/L, Cr 201.5mmol /L), and elevated levels of bilirubin (total bilirubin, 27.32 μmol /L; direct bilirubin, 8.55 μmol/L), ALP (16.02 μkat/L), LDH (4.26 μkat/L), CRP (340 mg/L), fibrinogen (13.31 g/L), and ferritin (1802 μg/L). The percentage of reticulated platelets was 16.8%. The prothrombin time was 14.3 sec (ref 11.5 sec), and the activated partial thromboplastin time was 47.1 sec (ref 29.6 sec). Rheumatoid factor, antinuclear antibodies, myeloperoxidase-anti-neutrophil cytoplasmic antibody (ANCA), proteinase3-ANCA, and anti-glomerular basement membrane antibody were normal. The serum IgG concentration was 6.94 g/L. Repeat blood cultures, β-D glucan, candida, aspergillus, CMV antigenemia, ADV, and Epstein-Barr virus-DNA were all negative. Bone marrow examination showed a normocellular marrow with <5% blast cells and the percentage of donor cells was 99.8%. Reticulin myelofibrosis and increased numbers of megakaryocytes were not observed. No morphological atypical megakaryocytes were noted. Computed tomography (CT) images showed multiple enlarged mediastinal lymph nodes and abdominal para-aortic lymph nodes that were approximately 1-1.5 cm in diameter. He also had gallstones, mild splenomegaly, bilateral pleural effusion, and pelvic ascites; however, there were no signs of infection. Upper endoscopic examination showed erosive gastritis and gastric ulcer, and a colonoscopic examination showed redness of the mucosa from the descending colon to the rectum. The diagnosis of gastrointestinal GVHD was made by biopsies from the body and fundus, although the patient’s clinical condition could not be explained by gastrointestinal GVHD alone. The severity of chronic GVHD was moderate according to the National Institutes of Health (NIH) criteria updated and published in 2015 [4].

**Figure 1:**
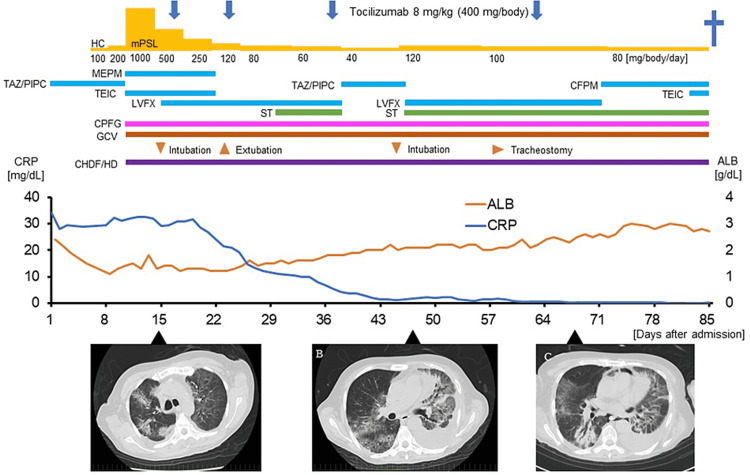
Clinical course of the patient. The graphs display the C-reactive protein values and albumin values, and the treatment interventions during the hospital course. (A) Computed tomography (CT) image taken on day 15 after admission shows bilateral ground-glass opacities and consolidations with a small amount of pleural effusion. (B) CT image taken on day 47 after admission shows extensive pneumonia and increased left pleural effusion. (C) CT image taken on day 68 after admission shows interstitial pneumonia with mediastinal emphysema and worsened systemic edema. HC, hydrocortisone; mPSL, methylprednisolone; TAZ/PIPC, tazobactam/piperacillin; MEPM, meropenem; TEIC, teicoplanin; LVFX, levofloxacin; CFPM, cefepime; ST, sulfamethoxazole trimethoprim; CPFG, caspofungin; GCV, ganciclovir; CHDF, continuous hemodiafiltration; HD, hemodialysis.

The fever and inflammatory response did not improve despite the introduction of 1 mg/kg (60 mg/body) of mPSL, and respiratory failure gradually worsened. Infections were unlikely, and pulmonary edema with high inflammation and non-infectious lung disorder were suspected. After progression of respiratory and kidney failure, the patient received multidisciplinary treatment with ventilator and continuous hemodiafiltration at the intensive care unit (ICU). The N-terminal-pro-brain natriuretic peptide level increased to 2,004 pg/mL at admission; however, left ventricular asynergy and valvular disease were not observed by transthoracic echocardiography (shown in Fig. 1).

Although a lymph node biopsy could not be performed because of thrombocytopenia and technical difficulties, the patient was clinically diagnosed with TAFRO syndrome according to the 2015 diagnostic criteria [2]. The disease severity on admission was severe (Grade 4) according to a minor update in the 2019 version [5]. The serum IL-6 level was 56.9 pg/mL (normal range, 0-4 pg/mL), the human herpesvirus 8 DNA value was <2.0 × 10^2^ copies/mL, and vascular endothelial growth factor (VEGF) level was normal after steroid therapy (29 pg/mL, normal range 0-115). mPSL pulse therapy was ineffective. Subsequently, tocilizumab, the anti-IL-6 receptor antibody, was administered twice a week at a dose of 8 mg/kg (400 mg/body); the CRP values were normalized, and the ventilator was removed nine days after intubation, although oxygen and steroids were not discontinued. At four days after extubation, the patient experienced confusion, and magnetic resonance imaging of the head showed subcortical cerebral infarction of the left temporal lobe. He received conservative treatment and left the ICU 22 days after extubation; however, respiratory failure recurred. Blood tests revealed elevated levels of serum IL-6 (937 pg/mL), KL-6 (1070 U/mL; normal range: <500 U/mL), and SP-D (498 U/mL; normal range: <110 ng/mL). CT images showed interstitial pneumonia with mediastinal emphysema and worsened systemic edema. Re-intubation was performed, and tocilizumab was re-administered with increasing doses of mPSL. A tracheostomy was performed for continuous respiratory management; however, the patient died two years and 10 months (day 1,034) after CBT because of respiratory failure. Permission for a post-mortem autopsy was not provided.

## Discussion

TAFRO syndrome is a heterogeneous clinical entity, which is derived from multiple potential causes including infectious diseases, malignancies, rheumatologic disorders, and iMCD [1,3]. The diagnostic criteria were first established in 2015; however, there was no mention of a relationship with HCT [2]. To the best of our knowledge, this is the first case report of post-transplant complications with TAFRO features in an AML patient who received CBT.

In the present case, a lymph node biopsy could not be performed. According to the 2015 diagnostic criteria [2], the patient meets three essential diagnostic criteria (anasarca, thrombocytopenia, and systemic inflammation) and two or more minor diagnostic criteria (mild organomegaly and progressive renal insufficiency). However, pathological data are critical for the definitive diagnosis of TAFRO syndrome in cases with a variety of complications, such as after allo-HCT. For a diagnosis of TAFRO syndrome after allo-HCT, it is important to exclude infectious diseases, GVHD, cGVHD-associated serositis, and recurrence of hematological disorders; however, this is often difficult. TAFRO syndrome is a systemic inflammatory disorder of unknown etiology, and GVHD is a systemic inflammatory condition mediated by the transplanted immune system. Post-transplant TAFRO symptoms may be a subtype of GVHD.

In this case, the onset of TAFRO symptoms was two years and seven months after CBT and one year after discontinuation of immunosuppressants. Treatment strategies for TAFRO syndrome include steroid pulse therapy, tocilizumab, tacrolimus, cyclosporine A, and rituximab [6-8]. Fujimoto et al. analyzed 68 patients diagnosed with TAFRO syndrome who received corticosteroid therapy as the first-line treatment; the second-line treatment was tocilizumab in 21, cyclosporine A in 14, and rituximab in eight patients in addition to corticosteroids. In the present case, as first-line treatment for GVHD, 1 mg/kg of mPSL was initiated, but it was ineffective. Subsequent mPSL pulse therapy was also unsuccessful. Because the patient had a strong inflammatory response, was refractory to steroid treatment, and was atypical for chronic GVHD, tocilizumab was administered based on suspicion of post-transplant TAFRO syndrome. Calcineurin inhibitors were also considered but were difficult to use due to renal insufficiency. Non-infectious lung injury, pulmonary edema, and cerebral infarction associated with TAFRO syndrome affected the disease course.

Elevated levels of IL-6 and/or VEGF were reported previously in patients with TAFRO syndrome [2]. IL-6 is a pro-inflammatory cytokine, and it induces synthesis of acute phase proteins such as CRP, serum amyloid A, fibrinogen, and hepcidin in hepatocytes [9]. On the other hand, it inhibits the production of albumin. Hypoalbuminemia associated with inflammation causes systemic edema and intravascular dehydration. Cerebral infarction in a patient with TAFRO syndrome was reported previously [10]. Increased IL-6 levels are also associated with severe pulmonary toxicity [11] and with reduced survival after autologous HCT [12]. Chen et al. reported that inhibition of the IL-6 signaling pathway by antibody-mediated blockade of the IL-6 receptor markedly reduces the number of Th1 and Th17 cells in GVHD target organs, thereby decreasing the pathological damage attributable to GVHD [13].

## Conclusions

In conclusion, we reported a fatal case of TAFRO syndrome-like complications that developed two years and seven months after CBT and one year after discontinuation of immunosuppressants. Tocilizumab showed efficacy in these symptoms, albeit for a short period of time. Investigating the frequency of TAFRO symptoms after allo-HCT and establishing a diagnostic strategy to distinguish TAFRO syndrome from GVHD is important to designing effective treatments.
